# Supplementary material for: Burden of drug-resistant tuberculosis among contacts of index cases: a protocol for a systematic review
Source: BMJ Open. 2024 Jan 9;14(1):e074364. doi: 10.1136/bmjopen-2023-074364 (PMC10806946; doi:10.1136/bmjopen-2023-074364)
Supplement: Author's manuscript [file bmjopen-2023-074364.draft_revisions.pdf]

BMJ Open

BMJ Open is committed to open peer review. As part of this commitment we make the peer review history of every article we publish publicly available.

When an article is published we post the peer reviewers' comments and the authors' responses online. We also post the versions of the paper that were used during peer review. These are the versions that the peer review comments apply to.

The versions of the paper that follow are the versions that were submitted during the peer review process. They are not the versions of record or the final published versions. They should not be cited or distributed as the published version of this manuscript.

BMJ Open is an open access journal and the full, final, typeset and author-corrected version of record of the manuscript is available on our site with no access controls, subscription charges or pay-per-view fees (http://bmjopen.bmj.com).

If you have any questions on BMJ Open's open peer review process please email info.bmjopen@bmj.com

BMJ Open

Burden of drug-resistant tuberculosis among contacts of index cases: a protocol for a systematic review

Journal:	*BMJ Open*
Manuscript ID	bmjopen-2023-074364
Article Type:	Protocol
Date Submitted by the Author:	13-Apr-2023

Complete List of Authors:	Akalu, Temesgen ; University of Gondar, Epidemiology and Biostatistics; Curtin University Faculty of Health Sciences,<br>Clements, Archie C. A.; Telethon Kids Institute; University of Plymouth<br>Gebreyohannes, Eyob Alemayehu; Telethon Kids Institute; University of Western Australia<br>Wolde, Haileab Fekadu; University of Gondar; Telethon Kids Institute<br>Shiferaw, Fasil Wagnaw; Australian National University<br>Alene, Kefyalew; Curtin University Bentley Campus, Health Science ; Telethon Kids Institute,

Keywords:	Systematic Review, Epidemiology < TROPICAL MEDICINE, TROPICAL MEDICINE

SCHOLARONE™
Manuscripts

Burden of drug-resistant tuberculosis among contacts of index cases: a protocol for a systematic review

Temesgen Yihunie Akalu[1,2,3*], Archie C. A. Clements[3, 4], Eyob Alemayehu Gebreyohannes[3,5], Haileab Fekadu Wolde[1,2,3], Fasil Wagnaw Shiferaw[6], Kefyalew Addis Alene[1, 2, 3]

Affiliations

[1]Institute of Public Health, College of Medicine and Health Sciences, University of Gondar, Gondar, Ethiopia

[2]School of Population Health, Faculty of Health Sciences, Curtin University, Australia

[3]Geospatial and Tuberculosis Research Team, Telethon Kids Institute, Australia

[4]Penninsula Medical School, University of Plymouth, Plymouth, United Kingdom

[5]School of Allied Health, University of Western Australia, Perth, Western Australia, Australia

[6]Australian National University, Canberra, Australian capital territory Australia

TYA: t.akalu@postgrad.curtin.edu.au

ACAC: archie.clements@plymouth.ac.uk

EAG: Eyob.Gebreyohannes@telethonkids.org.au

HFW: h.wolde@postgrad.curtin.edu.au

FWS: FasilWagnew.Shiferaw@anu.edu.au

KAA: FasilWagnew.Shiferaw@anu.edu.au

Corresponding author*: Temesgen Yihunie Akalu

Email: t.akalu@postgrad.curtin.edu.au

Address: 1 Kyle Avenue, Bentley WA, Australia 6102

Mobile: +61434340860

For peer review only - http://bmjopen.bmj.com/site/about/guidelines.xhtml

Abstract

Introduction: People having close contact with drug-resistant tuberculosis (DR-TB) patients are at increased risk of contracting and developing the disease. However, no comprehensive review has been undertaken to estimate the burden of active and latent DR-TB among contacts of DR-TB patients. Therefore, the current systematic review will quantify the prevalence and incidence of active and latent DR-TB among contacts of DR-TB patients.

Method and analysis: Systematic searches will be conducted in Medline, Embase, Web of Science, Scopus, Cochrane Central Register of Controlled trials (CENTRAL), and Cumulative Index to Nursing and Allied Health Literature (CINHAL) databases. The search will be conducted without restrictions on time, language, and geography. A random-effects meta-analysis will be conducted for effect estimates. The pooled prevalence and incidence of DR-TB will be compared between people with and without contact with DR-TB patients. We will also estimate an odds ratio or relative risk associated with direct contact. The presence of heterogeneity between studies will be assessed by Higgins I^2 statistics. Sub-group analysis and meta-regression will be conducted to determine the source of heterogeneity. The risk of bias will be assessed using a visual inspection of the funnel plot and Egger's regression test statistics. Trim and fill analysis will be done in the presence of publication bias. A sensitivity analysis will be conducted by trimming low-quality studies. The systematic review will be reported according to Preferred Reporting Items for Systematic Reviews and Meta-Analyses (PRISMA-20).

Ethics and dissemination: Ethical approval will not be required for this study as it will be a systematic review and meta-analysis based on previously published evidence. The findings of the systematic review will be presented at scientific conferences and published in scientific journals.

For peer review only - http://bmjopen.bmj.com/site/about/guidelines.xhtml

Protocol registration: The protocol is published in PROSPERO with registration number CRD42023390339.

Keywords: Contacts, drug-resistant tuberculosis, systematic review, protocol

Background

Drug-resistant tuberculosis (DR-TB) is an important public health concern. It is defined as resistance to any of the anti-TB drugs, and it can be classified into mono-resistant (resistant to only one anti-TB drug), multi-drug-resistant tuberculosis (MDR-TB: resistant to both isoniazid and rifampicin), poly-resistant (resistant to more than two first-line drugs except combined resistance to both isoniazid and rifampin), pre-XDR-TB (MDR-TB with resistance to either a fluoroquinolone, or at least 1 of 3 injectable second-line TB drugs, but not both), and extensively drug-resistant (XDR-TB: MDR-TB with resistance to any fluoroquinolone and at least one of the second-line injectable drugs) (1). In 2021, approximately half a million people were diagnosed with DR-TB and nearly 3.9% of new TB cases and 20% of previously treated cases were DR-TB. Three countries alone carry 42% of the global DR-TB burden in 2021: India (26%), the Russian Federation (8.5%), and Pakistan (7.9%) (2).

Contact investigation is an active case detection approach among contacts of drug-susceptible TB (DS-TB) and DR-TB patients and its primary is to foster early diagnosis and treatment. This will interrupting disease transmission, slowing down the progression of the disease, preventing long-term irreversible physical and mental health complications, as well as social, quality of life and financial harms, and reducing the overall mortality from DR-TB (3-5). The treatment of MDR-TB is costly, toxic, and takes an average treatment duration of two years (6, 7). Active case finding is recommended for people having a history of exposure to DR-TB cases as they are at a higher risk of developing the disease than the general population (8). However, the probability of developing

For peer review only - http://bmjopen.bmj.com/site/about/guidelines.xhtml

DR-TB among contacts will vary and depends on the infectiousness of the index case (9), duration of contact (9), proximity to the index case (10), and susceptibility of the contact (11). As a result, the timing of the disease occurrence among contacts varies from as short as six weeks to several years (12).

High-income countries, where the incidence of DR-TB is low in the general population, have standard practices regarding DR-TB contact investigation (13). Approaches including radiological investigation, sputum culture, drug susceptibility tests (DST), and sophisticated genomic methods (e.g., targeted next-generation sequencing (tNGS)) are used in identifying active DR-TB cases among contacts of DR-TB (14, 15). Tuberculin skin test (TST) and interferon-gamma tests are used in latent TB case detection (16, 17). However, DR-TB contact screening among contacts of DR-TB patients is very limited in low-income countries due to scarce resources, where the incidence of DS-TB and DR-TB is high (18). Recently, a growing interest in contact screening practices among contacts of DR-TB patients in low-income countries has been reported (19).

Several systematic reviews have estimated the burden of DS-TB among people who were close contacts of DS-TB cases. Those studies showed that people having close contact with DR-TB patients are at increased risk of contracting and developing the disease. For example, a previous systematic review conducted in high-income countries in 2005 by Morrison et al. showed that the overall burden of TB (both DS-TB and DR-TB) among contacts was 4.5%. However, the study lacked a stratified analysis of high-risk groups such as DR-TB close contacts and addressed only the prevalence of TB overall (20). Another systematic review conducted in low-income countries in 2013 by Fox et al. among contacts of TB patients (DS-TB and DR-TB combined) showed that the overall prevalence of active TB was 3.1% (4). The findings from previous studies have provided inconclusive evidence and are now outdated (21). Therefore, the current systematic

For peer review only - http://bmjopen.bmj.com/site/about/guidelines.xhtml

review will quantify the burden of DR-TB among people in contact with DR-TB patients including household, close, and casual contacts of DR-TB patients. The primary objective is to quantify the pooled proportion of active and latent DR-TB among people in close contact with DR-TB patients. Our secondary objective is to assess study-level characteristics that may be associated with a high proportion of DR-TB.

Review questions

What is the burden of latent DR-TB cases among contacts of index cases?

What is the burden of active DR-TB cases among contacts of index cases?

What is the risk of developing DR-TB among close contacts?

What are the levels of adherence, treatment outcomes, and adverse drug reactions among contacts of DR-TB cases?

Methods

Protocol registration

The protocol for this systematic review is registered in PROSPERO with a protocol registration number CRD42023390339 and reported according to the Preferred Reporting Items for Systematic Reviews and Meta-Analyses Protocols (PRISMA-P) statement 2015 (22). The article screening and selection processes will be reported using the PRISMA-20 flow chart **(Supplementary file 1)**.

Search strategy

Systematic searches will be conducted in Medline (Via OVID), Embase, Web of Science, Scopus, and Cumulative Index to Nursing and Allied Health Literature (CINAHL) databases. We will use

For peer review only - http://bmjopen.bmj.com/site/about/guidelines.xhtml

the Cochrane Central Register of Controlled Trials (CENTRAL) database to search for experimental and quasi-experimental studies. Other search engines such as Google and Google Scholar will be searched for grey literature. The search will be conducted from the inception of each database without restrictions on time, language, and geography. We will also perform hand-searching of the reference lists of included studies. When additional information is required, we will contact the corresponding authors. The search strategy for Medline is summarized in **Table 1**.

Table 1: Proposed search strategy in Medline

Search	Query
#1	(“multidrug-resistant* tuberculosis” or “multidrug-resistant* TB” or “extensively drug-resistant*” or “drug-resistant* tuberculosis” or “MDR-TB” or “XDR-TB” or DR-TB”).mp. [mp=title, book title, abstract, original title, name of substance word, subject heading word, floating sub-heading word, keyword heading word, organism supplementary concept word, protocol supplementary concept word, rare disease supplementary concept word, unique identifier, synonyms]
#2	("tracing" or "contact*" or "investigation" or "household" or "screening" or "infectious disease contact screening" or "household contact" or "close contact*" or "partner notification*" or "index case*").mp. [mp=title, book title, abstract, original title, name of substance word, subject heading word, floating sub-heading word, keyword heading word, organism supplementary concept word, protocol supplementary concept word, rare disease supplementary concept word, unique identifier, synonyms]
#3	1 AND 2

For peer review only - http://bmjopen.bmj.com/site/about/guidelines.xhtml

Eligibility criteria: All studies reporting the burden (i.e., proportion, prevalence, or incidence) of DR-TB among people with contacts (i.e., households, close, and casual contacts) of DR-TB will be included in this systematic review and meta-analysis. We will exclude reviews, commentaries, editorials, case reports and case series, and animal studies. Moreover, studies that lack information on the outcome variable and are conducted only on DS-TB patients will be excluded. Studies will be included based on the PICO (Population, Intervention, Comparator, and Outcome) framework.

Outcome measures

Primary outcome measures

The study's primary outcomes are the prevalence and incidence of latent and active DR-TB among people having contact with DR-TB patients. The incidence of DR-TB among people having contact with DR-TB patients will be calculated by year of enrolment. The prevalence or incidence of active DR-TB among people having contact with DR-TB will be determined. Contact will be defined as a person living in the same household as the index case or exposure with DR-TB patients in transportation, workplace, and recreational sites. Latent DR-TB will be taken in our study as defined by the original papers.

Secondary outcomes

Secondary outcomes will include levels of adherence, treatment side effects, and treatment outcomes among DR-TB patients identified through contact screening.

Study selection and data extraction

After a comprehensive search, data will be imported to Endnote version X7.8 (THOMSON REUTERS), and duplicates will be removed. Studies will be exported to Rayyan for screening by title and abstract. Two independent reviewers (TYA and HFW) will screen the title, abstract, and full texts to identify eligible studies. Any inconsistencies will be resolved through independent

For peer review only - http://bmjopen.bmj.com/site/about/guidelines.xhtml

screening by a third reviewer (FWS). TYA will prepare the data extraction checklist, and data will be extracted in a Microsoft Excel (version 365) spreadsheet. The following data will be extracted from the included studies: 1) Bibliographic details: name of the first author, year of publication, year of data collection, country, and World Health Organization (WHO) regions, 2) demographic characteristics of participants: mean/median age, the proportion of males, residence (urban vs rural), and the country's wealth status, 3) study characteristics: study design, sample size, type of drug-resistant tuberculosis, comorbidities like HIV and diabetes mellitus, the total number of people examined for DR-TB by gene Xpert, Line Prob Assay (LPA), and/or culture, the timing of developing DR-TB, frequency of contact, and location of contact (household, work place, child-care, and homeless), type of contacts (households, close, and casual), proportions of latent MDR- and XDR-TB, active MDR- and XDR-TB, overall proportions of latent MDR- and XDR-TB, and overall active MDR- and XDR-TB). Moreover, the index case characteristics (number, sex, median/mean age, comorbidities, and treatment categories) will be extracted. For a study done in multiple countries, the data from each country will be reported independently if available. The study screening and selection process is summarized in **Fig 1.**

Figure 1: Preferred Reporting Items for Systematic Reviews and Meta-analyses (PRISMA)-2020 flow diagram for the summary of the systematic review study selection process

Quality Assessment

The Newcastle-Ottawa quality assessment scale will be used to assess the quality of cohort and case-control studies (23). The quality of cross-sectional studies will be assessed using the modified version of the Newcastle-Ottawa Quality Assessment Scale (24). The score will classify studies

For peer review only - http://bmjopen.bmj.com/site/about/guidelines.xhtml

into low-quality (a score between 1 and 4), moderate quality (a score between 5 and 7), and high-quality studies (a score between 8 and 9). To assess the quality of interventional studies, the Cochrane Handbook for Systematic Reviews of Interventions (Version 6.3, 2022) will be used (25). The quality of the included studies will be done by the two reviewers (TYA and FWS). A third reviewer (HFW) will be involved to resolve any disagreements between the two primary reviewers. Moreover, the Meta-analysis Of Observational Studies in Epidemiology (MOOSE) statement will be used for reporting the results of the systematic review and meta-analysis (26).

Data synthesis and analysis

We are interested in estimating the burden of latent and active DR-TB reported as incidence or prevalence at the global level. Stata version 17 software will be used to conduct the analysis. For incidence studies, the incidence rate will be calculated as the number of incident cases per year divided by the population at risk. Similarly, for the prevalence study, the prevalence will be calculated as the number of prevalent cases divided by the total population and expressed as a proportion. A forest plot will be generated to show individual and pooled prevalence of latent and/or active DR-TB cases among DR-TB contacts, 95% confidence interval (CI), name of the first author, publication years, and study weights. A random-effects meta-analysis will be used to report the pooled estimates. The presence of heterogeneity among the included studies will be evaluated using the I^2 statistics and a 95% CI. An I^2 value close to zero indicates no observed heterogeneity and a larger value of I^2 shows an increased level of heterogeneity. Heterogeneity will be considered low, moderate, and high when the values are below 25%, between 25% and 75%, and above 75%, respectively (27). To identify the source of heterogeneity, sub-group analysis will be carried out by study characteristics. Moreover, meta-regression will be conducted to assure the existing source of heterogeneity. Publication bias will be assessed visually using

For peer review only - http://bmjopen.bmj.com/site/about/guidelines.xhtml

funnel plots and statistically using Egger's regression test. A trim and fill analysis will be conducted as an adjustment if there is any publication bias (28). A sensitivity analysis will be done by trimming low-quality studies.

Implication of the review

DR-TB contact investigation is a top priority in DR-TB infection control, being critical for locating the source of infections as patients with smear-positive DR-TB are highly contagious. Identification of cases through contact investigation can lead to timely treatment and preventative measures to be undertaken, thereby minimizing the risk of disease transmission, and further reducing the burden of DR-TB in the general population. Early diagnosis and detecting of DR-TB will improve treatment outcomes and reduce adverse drug reactions and complications. It will also reduce cost of the patients and households. Overall, the study will help to achieve the three END-TB targets 0f 2035 (no catastrophic cost, 90% reduction in mortality, and 95% reduction in patients suffering from TB) through early diagnosis and treatment.

Article summary

Strength and limitation

- To the best of our knowledge, there is limited and outdated evidence on the burden of DR-TB among their contacts.
- This study will determine the burden of XDR-TB among contacts of DR-TB patients which is not in previous systematic reviews.
- The study will not be representative of the 30 high-burden TB countries due to the scarcity of published articles in some high-burden countries.
- Heterogeneity among included studies may be the other limitation of the study.

For peer review only - http://bmjopen.bmj.com/site/about/guidelines.xhtml

Declaration

Acknowledgments

We would like to acknowledge Curtin University for financial funding.

Author contributions

TYA designed the study and wrote the initial draft of the manuscript. ACAC, EAG, HFW, FWS, and KAA critically reviewed the final manuscript. All authors approved the final manuscript for submission.

Patient and public involvement

Patients were not involved in the development of the research question, study design, and outcome measures.

Ethics and dissemination

Ethical approval will not be required for this study as it will be a systematic review and meta-analysis based on previously published studies. In addition to scientific publication, the results will be disseminated on social media platforms including Twitter and LinkedIn to inform policymakers, funders, and other researchers.

Funding

This work is supported by the Australian National Health and Medical Research Council (NHMRC) through an Emerging Leadership Investigator Grant APP1196549. KAA is a senior researcher at Curtin University who received the fund. TYA is also supported by Curtin University

For peer review only

For peer review only - http://bmjopen.bmj.com/site/about/guidelines.xhtml

Higher Degree Research (HDR) Scholarship and acknowledges Curtin University for providing support. However, the funders had no role in design, analysis, and interpretations of findings.

Competing interest

The authors declare that they have no conflicts of interest.

Patient consent for publication

Not required

Data sharing statement

Data will be available upon a reasonable request from the corresponding author.

References

1. Seddon JA, Perez-Velez CM, Schaaf HS, Furin JJ, Marais BJ, Tebruegge M, et al. Consensus statement on research definitions for drug-resistant tuberculosis in children. Journal of the Pediatric Infectious Diseases Society. 2013;2(2):100-9.
2. WHO. Global tuberculosis report 2022. Geneva: World Health Organization; 2022.
3. Rieder H. Contacts of tuberculosis patients in high-incidence countries. The international journal of tuberculosis and lung disease. 2003;7(12):S333-S6.
4. Fox GJ, Barry SE, Britton WJ, Marks GB. Contact investigation for tuberculosis: a systematic review and meta-analysis. Eur Respir J. 2013;41(1):140-56.
5. Cook VJ, Sun SJ, Tapia J, Muth SQ, Argüello DF, Lewis BL, et al. Transmission network analysis in tuberculosis contact investigations. The Journal of infectious diseases. 2007;196(10):1517-27.
6. Seung KJ, Omatayo DB, Keshavjee S, Furin JJ, Farmer PE, Satti H. Early outcomes of MDR-TB treatment in a high HIV-prevalence setting in Southern Africa. PloS one. 2009;4(9):e7186.
7. Orenstein EW, Basu S, Shah NS, Andrews JR, Friedland GH, Moll AP, et al. Treatment outcomes among patients with multidrug-resistant tuberculosis: systematic review and meta-analysis. The Lancet infectious diseases. 2009;9(3):153-61.
8. Greenaway C, Palayew M, Menzies D. Yield of casual contact investigation by the hour. The International Journal of Tuberculosis and Lung Disease. 2003;7(12):S479-S85.
9. Fok A, Numata Y, Schulzer M, FitzGerald M. Risk factors for clustering of tuberculosis cases: a systematic review of population-based molecular epidemiology studies. The International Journal of Tuberculosis and Lung Disease. 2008;12(5):480-92.

For peer review only

For peer review only - http://bmjopen.bmj.com/site/about/guidelines.xhtml

10. Kenyon TA, Valway SE, Ihle WW, Onorato IM, Castro KG. Transmission of multidrug-resistant Mycobacterium tuberculosis during a long airplane flight. New England Journal of Medicine. 1996;334(15):933-8.
11. YIM JJ, Selvaraj P. Genetic susceptibility in tuberculosis. Respirology. 2010;15(2):241-56.
12. Marks SM, Taylor Z, Qualls NL, Shrestha-Kuwahara RJ, Wilce MA, Nguyen CH. Outcomes of contact investigations of infectious tuberculosis patients. American journal of respiratory and critical care medicine. 2000;162(6):2033-8.
13. Society JTCotBT. Control and prevention of tuberculosis in the United Kingdom: code of practice 2000. Thorax. 2000;55(11):887-901.
14. Cabibbe AM, Spitaleri A, Battaglia S, Colman RE, Suresh A, Uplekar S, et al. Application of Targeted Next-Generation Sequencing Assay on a Portable Sequencing Platform for Culture-Free Detection of Drug-Resistant Tuberculosis from Clinical Samples. J Clin Microbiol. 2020;58(10).
15. Hoek KG, Van Rie A, van Helden PD, Warren RM, Victor TC. Detecting drug-resistant tuberculosis: the importance of rapid testing. Molecular Diagnosis & Therapy. 2011;15:189-94.
16. Teixeira L, Perkins M, Johnson J, Keller R, Palaci M, do Valle Dettoni V, et al. Infection and disease among household contacts of patients with multidrug-resistant tuberculosis. The International Journal of Tuberculosis and Lung Disease. 2001;5(4):321-8.
17. Diel R, Loddenkemper R, Meywald-Walter K, Gottschalk R, Nienhaus A. Comparative performance of tuberculin skin test, QuantiFERON-TB-Gold In Tube assay, and T-Spot. TB test in contact investigations for tuberculosis. Chest. 2009;135(4):1010-8.
18. Organization WH. Recommendations for investigating contacts of persons with infectious tuberculosis in low-and middle-income countries: World Health Organization; 2012.
19. Hwang T, Ottmani S, Uplekar M. A rapid assessment of prevailing policies on tuberculosis contact investigation. The International journal of tuberculosis and lung disease. 2011;15(12):1620-3.
20. Morrison J, Pai M, Hopewell PC. Tuberculosis and latent tuberculosis infection in close contacts of people with pulmonary tuberculosis in low-income and middle-income countries: a systematic review and meta-analysis. The Lancet infectious diseases. 2008;8(6):359-68.
21. Shah NS, Yuen CM, Heo M, Tolman AW, Becerra MC. Yield of contact investigations in households of patients with drug-resistant tuberculosis: systematic review and meta-analysis. Clin Infect Dis. 2014;58(3):381-91.
22. Moher D, Stewart L, Shekelle P. Implementing PRISMA-P: recommendations for prospective authors. Syst Rev. 2016;5:15.
23. Wells GA, Shea B, O'Connell D, Peterson J, Welch V, Losos M, et al. The Newcastle-Ottawa Scale (NOS) for assessing the quality of nonrandomised studies in meta-analyses. Oxford; 2000.
24. Norris JM, Simpson BS, Ball R, Freeman A, Kirkham A, Parry MA, et al. A modified Newcastle-Ottawa scale for assessment of study quality in genetic urological research. European Urology. 2021;79(3):325-6.
25. Higgins JP, Thomas J, Chandler J, Cumpston M, Li T, Page MJ, et al. Cochrane handbook for systematic reviews of interventions: John Wiley & Sons; 2019.
26. Stroup DF, Berlin JA, Morton SC, Olkin I, Williamson GD, Rennie D, et al. Meta-analysis of observational studies in epidemiology: a proposal for reporting. Jama. 2000;283(15):2008-12.
27. Schober P, Mascha EJ, Vetter TR. Statistics from a (agreement) to Z (z score): a guide to interpreting common measures of association, agreement, diagnostic accuracy, effect size, heterogeneity, and reliability in medical research. Anesthesia & Analgesia. 2021;133(6):1633-41.
28. Zhai J, Mu W, Si J, Li Y, Zhao C, Shang H, et al. Acupuncture for constipation in patients with stroke: protocol of a systematic review and meta-analysis. BMJ open. 2018;8(3):e020400.

For peer review only

For peer review only - http://bmjopen.bmj.com/site/about/guidelines.xhtml

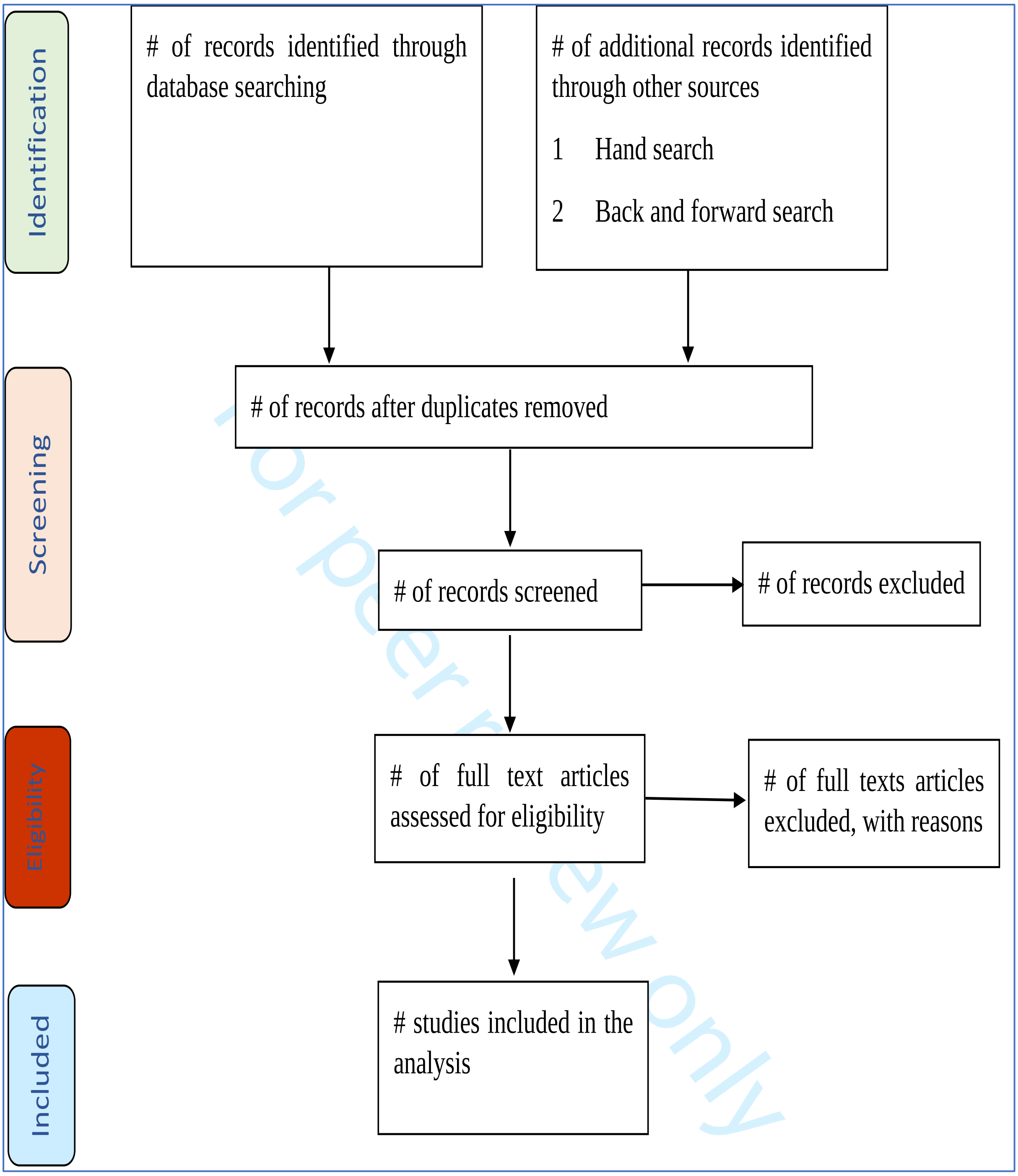

Figure 1: Preferred Reporting Items for Systematic Reviews and Meta-analyses (PRISMA)-2020 flow diagram for the summary of the systematic review study selection process

For peer review only

Reporting checklist for protocol of a systematic review and meta-analysis.

Based on the PRISMA-P guidelines.

Instructions to authors

Complete this checklist by entering the page numbers from your manuscript where readers will find each of the items listed below.

Your article may not currently address all the items on the checklist. Please modify your text to include the missing information. If you are certain that an item does not apply, please write "n/a" and provide a short explanation.

Upload your completed checklist as an extra file when you submit to a journal.

In your methods section, say that you used the PRISMA-Preporting guidelines, and cite them as:

Moher D, Shamseer L, Clarke M, Ghersi D, Liberati A, Petticrew M, Shekelle P, Stewart LA. Preferred Reporting Items for Systematic Review and Meta-Analysis Protocols (PRISMA-P) 2015 statement. Syst Rev. 2015;4(1):1.

		Reporting Item	Page Number
**Title**			
Identification	#1a	Identify the report as a protocol of a systematic review	1
Update	#1b	If the protocol is for an update of a previous systematic review, identify as such	NA

For peer review only

**Registration**			
	#2	If registered, provide the name of the registry (such as PROSPERO) and registration number	2
**Authors**			
Contact	#3a	Provide name, institutional affiliation, e-mail address of all protocol authors; provide physical mailing address of corresponding author	1
Contribution	#3b	Describe contributions of protocol authors and identify the guarantor of the review	3
**Amendments**			
	#4	If the protocol represents an amendment of a previously completed or published protocol, identify as such and list changes; otherwise, state plan for documenting important protocol amendments	
**Support**			
Sources	#5a	Indicate sources of financial or other support for the review	12
Sponsor	#5b	Provide name for the review funder and / or sponsor	12
Role of sponsor or funder	#5c	Describe roles of funder(s), sponsor(s), and / or institution(s), if any, in developing the protocol	12
**Introduction**			
Rationale	#6	Describe the rationale for the review in the context of what is	3-4

For peer review only

		already known	
Objectives	#7	Provide an explicit statement of the question(s) the review will address with reference to participants, interventions, comparators, and outcomes (PICO)	4
**Methods**			
Eligibility criteria	#8	Specify the study characteristics (such as PICO, study design, setting, time frame) and report characteristics (such as years considered, language, publication status) to be used as criteria for eligibility for the review	7
Information sources	#9	Describe all intended information sources (such as electronic databases, contact with study authors, trial registers or other grey literature sources) with planned dates of coverage	6
Search strategy	#10	Present draft of search strategy to be used for at least one electronic database, including planned limits, such that it could be repeated	6-7
Study records - data management	#11a	Describe the mechanism(s) that will be used to manage records and data throughout the review	9
Study records - selection process	#11b	State the process that will be used for selecting studies (such as two independent reviewers) through each phase of the review (that is, screening, eligibility and inclusion in meta-analysis)	8
Study records - data collection	#11c	Describe planned method of extracting data from reports (such as piloting forms, done independently, in duplicate), any	8

For peer review only

process		processes for obtaining and confirming data from investigators	
Data items	#12	List and define all variables for which data will be sought (such as PICO items, funding sources), any pre-planned data assumptions and simplifications	7
Outcomes and prioritization	#13	List and define all outcomes for which data will be sought, including prioritization of main and additional outcomes, with rationale	7
Risk of bias in individual studies	#14	Describe anticipated methods for assessing risk of bias of individual studies, including whether this will be done at the outcome or study level, or both; state how this information will be used in data synthesis	10
Data synthesis	#15a	Describe criteria under which study data will be quantitatively synthesised	9
Data synthesis	#15b	If data are appropriate for quantitative synthesis, describe planned summary measures, methods of handling data and methods of combining data from studies, including any planned exploration of consistency (such as I2, Kendall's τ)	9
Data synthesis	#15c	Describe any proposed additional analyses (such as sensitivity or subgroup analyses, meta-regression)	10
Data synthesis	#15d	If quantitative synthesis is not appropriate, describe the type of summary planned	NA
Meta-bias(es)	#16	Specify any planned assessment of meta-bias(es) (such as publication bias across studies, selective reporting within	10

For peer review only

		studies)	
Confidence in cumulative evidence	#17	Describe how the strength of the body of evidence will be assessed (such as GRADE)	10-11

None The PRISMA-P elaboration and explanation paper is distributed under the terms of the Creative Commons Attribution License CC-BY. This checklist can be completed online using https://www.goodreports.org/, a tool made by the EQUATOR Network in collaboration with Penelope.ai

For peer review only

BMJ Open

Burden of drug-resistant tuberculosis among contacts of index cases: a protocol for a systematic review

Journal:	*BMJ Open*
Manuscript ID	bmjopen-2023-074364.R1
Article Type:	Protocol
Date Submitted by the Author:	24-Oct-2023
Complete List of Authors:	Akalu, Temesgen ; University of Gondar, Epidemiology and Biostatistics; Curtin University Faculty of Health Sciences,<br>Clements, Archie C. A.; Telethon Kids Institute; University of Plymouth<br>Gebreyohannes, Eyob Alemayehu; Telethon Kids Institute; University of Western Australia<br>Wolde, Haileab Fekadu; University of Gondar; Telethon Kids Institute<br>Shiferaw, Fasil Wagnaw; Australian National University<br>Alene, Kefyalew; Curtin University Bentley Campus, Health Science ; Telethon Kids Institute,
**Primary Subject Heading**:	Infectious diseases
Secondary Subject Heading:	Epidemiology, Public health, Research methods
Keywords:	Systematic Review, Epidemiology < TROPICAL MEDICINE, TROPICAL MEDICINE

SCHOLARONE™
Manuscripts

Burden of drug-resistant tuberculosis among contacts of index cases: a protocol for a systematic review

Temesgen Yihunie Akalu[1,2,3*], Archie C. A. Clements[3, 4], Eyob Alemayehu Gebreyohannes[3,5], Haileab Fekadu Wolde[1,2,3], Fasil Wagnaw Shiferaw[6], Kefyalew Addis Alene[2, 3]

Affiliations

[1]Institute of Public Health, College of Medicine and Health Sciences, University of Gondar, Gondar, Ethiopia

[2]School of Population Health, Faculty of Health Sciences, Curtin University, Australia

[3]Geospatial and Tuberculosis Research Team, Telethon Kids Institute, Australia

[4]Penninsula Medical School, University of Plymouth, Plymouth, United Kingdom

[5]School of Allied Health, University of Western Australia, Perth, Western Australia, Australia

[6]Australian National University, Canberra, Australian capital territory Australia

TYA: temesgenyihunie@gmail.com

ACAC: archie.clements@plymouth.ac.uk

EAG: Eyob.Gebreyohannes@telethonkids.org.au

HFW: h.wolde@postgrad.curtin.edu.au

FWS: FasilWagnew.Shiferaw@anu.edu.au

KAA: FasilWagnew.Shiferaw@anu.edu.au

Corresponding author*: Temesgen Yihunie Akalu

Email: temesgenyihunie@gmail.com

Address: 1 Kyle Avenue, Bentley, WA, Australia 6102

Mobile: +61434340860

For peer review only

For peer review only - http://bmjopen.bmj.com/site/about/guidelines.xhtml

Abstract

Introduction: People having close contact with drug-resistant tuberculosis (DR-TB) patients are at increased risk of contracting and developing the disease. However, no comprehensive review has been undertaken to estimate the burden of DR-TB among contacts of DR-TB patients. Therefore, the current systematic review will quantify the prevalence and incidence of DR-TB among contacts of DR-TB patients.

Method and analysis: Systematic searches will be conducted in Medline, Embase, Web of Science, Scopus, Cochrane Central Register of Controlled trials (CENTRAL), and Cumulative Index to Nursing and Allied Health Literature (CINHAL) databases. The search will be conducted without restrictions on time, language, and geography. A random-effects meta-analysis will be conducted for effect estimates. The pooled prevalence and incidence of DR-TB will be compared between people with and without contact with DR-TB patients. The presence of heterogeneity between studies will be assessed by Higgins I^2 statistics. Sub-group analysis will be conducted to determine the source of heterogeneity. The risk of bias will be assessed using a visual inspection of the funnel plot and Egger's regression test statistics. Trim and fill analysis will be done in the presence of publication bias. A sensitivity analysis will be conducted by trimming low-quality studies. The systematic review will be reported according to Preferred Reporting Items for Systematic Reviews and Meta-Analyses (PRISMA-P) guideline.

Ethics and dissemination: Ethical approval will not be required for this study as it will be a systematic review and meta-analysis based on previously published evidence. The findings of the systematic review will be presented at scientific conferences and published in scientific journals.

Protocol registration: The protocol is published in PROSPERO with registration number CRD42023390339.

For peer review only - http://bmjopen.bmj.com/site/about/guidelines.xhtml

Keywords: Contacts, drug-resistant tuberculosis, systematic review, protocol

Article summary

Strength and limitation

- The review will use a comprehensive search strategy to obtain unbiased summary.
- Sub-group analysis will be performed to compare the prevalence and incidence of DR-TB by study characteristics.
- Findings will be reported according to Preferred Reporting Items for Systematic Reviews and Meta-Analyses protocol.
- The search will be conducted without time and geographical restriction.
- Substantial heterogeneity among included studies may be the possible limitation of the study.

Background

Drug-resistant tuberculosis (DR-TB) is an important public health concern. It is defined as resistance to any of the anti-TB drugs, and it can be classified into mono-resistant (resistant to only one anti-TB drug), multi-drug-resistant tuberculosis (MDR-TB: resistant to both isoniazid and rifampicin), poly-resistant (resistant to more than two first-line drugs except combined resistance to both isoniazid and rifampin), pre-XDR-TB (MDR-TB with resistance to either a fluoroquinolone, or at least 1 of 3 injectable second-line TB drugs, but not both), and extensively drug-resistant (XDR-TB: MDR-TB with resistance to any fluoroquinolone and at least one of the second-line injectable drugs) (1). In 2021, approximately half a million people were diagnosed with DR-TB and nearly 3.9% of new TB cases and 20% of previously treated cases were DR-TB.

For peer review only

For peer review only - http://bmjopen.bmj.com/site/about/guidelines.xhtml

Three countries alone carry 42% of the global DR-TB burden in 2021: India (26%), the Russian Federation (8.5%), and Pakistan (7.9%) (2).

Contact investigation is an active case detection approach among contacts of drug-susceptible TB (DS-TB) and DR-TB patients, and its primary is to foster early diagnosis and treatment. This will interrupt disease transmission, slowing down the progression of the disease, preventing long-term irreversible physical and mental health complications, as well as social, quality of life, and financial harms, and reducing the overall mortality from DR-TB (3-5). The treatment of MDR-TB is costly, toxic, and takes an average treatment duration of two years (6, 7). Active case finding is recommended for people having a history of exposure to DR-TB cases as they are at a higher risk of developing the disease than the general population (8). However, the probability of developing DR-TB among contacts will vary and depends on the infectiousness of the index case (9), duration of contact (9), proximity to the index case (10), and susceptibility of the contact (11). As a result, the timing of the disease occurrence among contacts varies from as short as six weeks to several years (12).

High-income countries, where the incidence of DR-TB is low in the general population, have standard practices regarding DR-TB contact investigation (13). Approaches including radiological investigation, sputum culture, drug susceptibility tests (DST), and sophisticated genomic methods (e.g., targeted next-generation sequencing (tNGS)) are used in identifying DR-TB cases among contacts of DR-TB (14, 15). Tuberculin skin test (TST) and interferon-gamma tests are used in latent TB case detection (16, 17). However, DR-TB contact screening among contacts of DR-TB patients is very limited in low-income countries due to scarce resources, where the incidence of DS-TB and DR-TB is high (18). Recently, a growing interest in contact screening practices among contacts of DR-TB patients in low-income countries has been reported (19).

For peer review only - http://bmjopen.bmj.com/site/about/guidelines.xhtml

Several systematic reviews have estimated the burden of DS-TB among people who were close contacts of DS-TB cases. Those studies showed that people having close contact with DR-TB patients are at increased risk of contracting and developing the disease. For example, a previous systematic review conducted in high-income countries in 2005 by Morrison et al. showed that the overall burden of TB (both DS-TB and DR-TB) among contacts was 4.5%. However, the study lacked a stratified analysis of high-risk groups such as DR-TB close contacts and addressed only the prevalence of TB overall (20). Another systematic review conducted in low-income countries in 2013 by Fox et al. among contacts of TB patients (DS-TB and DR-TB combined) showed that the overall prevalence of TB was 3.1% (4). The findings from previous studies have provided inconclusive evidence and are now outdated (21). Therefore, the current systematic review will quantify the burden of DR-TB among people in contact with DR-TB patients including household, close, and casual contacts of DR-TB patients. The primary objective is to quantify the pooled proportion of DR-TB among people in close contact with DR-TB patients. Our secondary objective is to assess study-level characteristics that may be associated with a high proportion of DR-TB.

Review questions

What is the prevalence of DR-TB among contacts of DR-TB patients?

What is the incidence of DR-TB among contacts of DR-TB patients?

What are the study level characteristics associated with high prevalence and incidences of DR-TB among contacts of DR-TB patients?

For peer review only - http://bmjopen.bmj.com/site/about/guidelines.xhtml

Methods

Protocol registration

The protocol for this systematic review is registered in PROSPERO with a protocol registration number CRD42023390339 and reported according to the Preferred Reporting Items for Systematic Reviews and Meta-Analyses Protocols (PRISMA-P) statement 2015 (22). The article screening and selection processes will be reported using the PRISMA-20 flow chart **(Supplementary file 1)**.

Search strategy

Systematic searches will be conducted in Medline (Via OVID), Embase, Web of Science, Scopus, and Cumulative Index to Nursing and Allied Health Literature (CINAHL) databases. We will use the Cochrane Central Register of Controlled Trials (CENTRAL) database to search for experimental and quasi-experimental studies. Other search engines such as Google and Google Scholar will be searched for grey literature. The search will be conducted from the inception of each database without restrictions on time and geography. We will also perform hand-searching of the reference lists of included studies. When additional information is required, we will contact the corresponding authors. The search strategy for Medline is summarized in **Table 1**.

Table 1: Proposed search strategy in Medline

**Search**	**Query**
#1	("multidrug-resistant* tuberculosis" or "multidrug-resistant* TB" or "extensively drug-resistant*" or "drug-resistant* tuberculosis" or "MDR-TB" or "XDR-TB" or DR-TB").mp. [mp=title, book title, abstract, original title, name of substance word, subject heading word, floating sub-heading word, keyword heading word, organism

For peer review only - http://bmjopen.bmj.com/site/about/guidelines.xhtml

	supplementary concept word, protocol supplementary concept word, rare disease supplementary concept word, unique identifier, synonyms]
#2	("tracing" or "contact*" or "investigation" or "household" or "screening" or "infectious disease contact screening" or "household contact" or "close contact*" or "partner notification*" or "index case*").mp. [mp=title, book title, abstract, original title, name of substance word, subject heading word, floating sub-heading word, keyword heading word, organism supplementary concept word, protocol supplementary concept word, rare disease supplementary concept word, unique identifier, synonyms]
#3	1 AND 2

Eligibility criteria: All studies reporting the burden (i.e., proportion, prevalence, or incidence) of DR-TB among people with contacts (i.e., households, close, and casual contacts) of DR-TB will be included in this systematic review and meta-analysis. We will exclude reviews, commentaries, editorials, case reports and case series, and animal studies. Moreover, studies that lack information on the outcome variable and are conducted only on DS-TB patients will be excluded. Studies will be included based on the PICO (Population, Intervention, Comparator, and Outcome) framework.

Outcome measures

Primary outcome measures

The primary outcomes of the study are the prevalence and incidence of DR-TB among people having contact with DR-TB patients. The incidence of DR-TB among people having contact with DR-TB patients will be calculated by year of enrolment. The prevalence or incidence of DR-TB among people having contact with DR-TB will be determined. Contact will be defined as a person

For peer review only

For peer review only - http://bmjopen.bmj.com/site/about/guidelines.xhtml

living in the same household as the index case or exposure to DR-TB patients in transportation, workplace, and recreational sites.

Study selection and data extraction

After a comprehensive search, data will be imported to Endnote version X7.8 (THOMSON REUTERS), and duplicates will be removed. Studies will be exported to Rayyan for screening by title and abstract. Two independent reviewers (TYA and EAG) will screen the title, abstract, and full texts to identify eligible studies. Any inconsistencies will be resolved through consensus between the two reviewers. TYA will prepare the data extraction checklist, and data will be extracted in a Microsoft Excel (version 365) spreadsheet. The following data will be extracted from the included studies: 1) Bibliographic details: name of the first author, year of publication, year of data collection, country, and World Health Organization (WHO) regions, 2) demographic characteristics of participants: mean/median age, the proportion of males, and the country's wealth status, 3) study characteristics: study design, sample size, type of drug-resistant tuberculosis, comorbidities like HIV and diabetes mellitus, the total number of people examined for DR-TB by gene Xpert, Line Prob Assay (LPA), and/or culture, the timing of developing DR-TB, frequency of contact, and location of contact (household, work place, child-care, and homeless), type of contacts (households, close, and casual), proportions of MDR- and XDR-TB,). For a study done in multiple countries, the data from each country will be reported independently if available. The study screening and selection process is summarized in **Fig 1.**

Figure 1: Preferred Reporting Items for Systematic Reviews and Meta-analyses (PRISMA)-2020 flow diagram for the summary of the systematic review study selection process

For peer review only

For peer review only - http://bmjopen.bmj.com/site/about/guidelines.xhtml

Quality Assessment

The Newcastle-Ottawa quality assessment scale will be used to assess the quality of retrospective and prospective cohort studies (23). The quality of cross-sectional studies will be assessed using the modified version of the Newcastle-Ottawa Quality Assessment Scale (24). The score will classify studies into low-quality (a score between 1 and 4), moderate quality (a score between 5 and 7), and high-quality studies (a score between 8 and 9). The quality of the included studies will be done by the two reviewers (TYA and EAG). Disagreements will be resolved by the consensus between the two reviewers.

Data synthesis and analysis

We are interested in estimating the burden of DR-TB reported as incidence or prevalence at the global level. Stata version 17 software will be used to conduct the analysis. For incidence studies, the incidence rate will be calculated as the number of incident cases per year divided by the population at risk. Similarly, for the prevalence study, the prevalence will be calculated as the number of prevalent cases divided by the total population and expressed as a proportion. A forest plot will be generated to show individual and pooled prevalence of DR-TB cases among DR-TB contacts, 95% confidence interval (CI), name of the first author, publication years, and study weights. A random-effects meta-analysis will be used to report the pooled estimates. The presence of heterogeneity among the included studies will be evaluated using the I^2 statistics and a 95% CI. An I^2 value close to zero indicates no observed heterogeneity and a larger value of I^2 shows an increased level of heterogeneity. Heterogeneity will be considered low, moderate, and high when the values are below 25%, between 25% and 75%, and above 75%, respectively (25). To identify the source of heterogeneity, sub-group analysis will be carried out by study characteristics. Moreover, meta-regression will be conducted to assure the existing source of heterogeneity.

For peer review only - http://bmjopen.bmj.com/site/about/guidelines.xhtml

Publication bias will be assessed visually using funnel plots and statistically using Egger's regression test. A trim and fill analysis will be conducted as an adjustment if there is any publication bias (26). A sensitivity analysis will be done by trimming low-quality studies.

Implication of the review

DR-TB contact investigation is a top priority in DR-TB infection control, being critical for locating the source of infections as patients with smear-positive DR-TB are highly contagious. Identification of cases through contact investigation can lead to timely treatment and preventative measures to be undertaken, thereby minimizing the risk of disease transmission, and further reducing the burden of DR-TB in the general population. Early diagnosis and detection of DR-TB will improve treatment outcomes and reduce adverse drug reactions and complications. It will also reduce the cost for the patients and households. Overall, the study will help to achieve the three END-TB targets of 2035 (no catastrophic cost, 90% reduction in mortality, and 95% reduction in patients suffering from TB) through early diagnosis and treatment.

Declaration

Acknowledgments

We would like to acknowledge Curtin University for financial funding.

Author contributions

TYA designed the study and wrote the initial draft of the manuscript. ACAC, EAG, HFW, FWS, and KAA critically reviewed the final manuscript. All authors approved the final manuscript for submission.

For peer review only - http://bmjopen.bmj.com/site/about/guidelines.xhtml

Patient and public involvement

Patients were not involved in the development of the research question, study design, and outcome measures.

Ethics and dissemination

Ethical approval will not be required for this study as it will be a systematic review and meta-analysis based on previously published studies. In addition to scientific publication, the results will be disseminated on social media platforms including Twitter and LinkedIn to inform policymakers, funders, and other researchers.

Funding

This work is supported by the Australian National Health and Medical Research Council (NHMRC) through an Emerging Leadership Investigator Grant APP1196549. KAA is a senior researcher at Curtin University who received the fund. TYA is also supported by Curtin University Higher Degree Research (HDR) Scholarship and acknowledges Curtin University for providing support. However, the funders had no role in design, analysis, and interpretations of findings.

Competing interest

The authors declare that they have no conflicts of interest.

Patient consent for publication

Not required

Data sharing statement

Data will be available upon a reasonable request from the corresponding author.

For peer review only

For peer review only - http://bmjopen.bmj.com/site/about/guidelines.xhtml

References

1. Seddon JA, Perez-Velez CM, Schaaf HS, Furin JJ, Marais BJ, Tebruegge M, et al. Consensus statement on research definitions for drug-resistant tuberculosis in children. Journal of the Pediatric Infectious Diseases Society. 2013;2(2):100-9.
2. WHO. Global tuberculosis report 2022. Geneva: World Health Organization; 2022.
3. Rieder H. Contacts of tuberculosis patients in high-incidence countries. The international journal of tuberculosis and lung disease. 2003;7(12):S333-S6.
4. Fox GJ, Barry SE, Britton WJ, Marks GB. Contact investigation for tuberculosis: a systematic review and meta-analysis. Eur Respir J. 2013;41(1):140-56.
5. Cook VJ, Sun SJ, Tapia J, Muth SQ, Argüello DF, Lewis BL, et al. Transmission network analysis in tuberculosis contact investigations. The Journal of infectious diseases. 2007;196(10):1517-27.
6. Seung KJ, Omatayo DB, Keshavjee S, Furin JJ, Farmer PE, Satti H. Early outcomes of MDR-TB treatment in a high HIV-prevalence setting in Southern Africa. PloS one. 2009;4(9):e7186.
7. Orenstein EW, Basu S, Shah NS, Andrews JR, Friedland GH, Moll AP, et al. Treatment outcomes among patients with multidrug-resistant tuberculosis: systematic review and meta-analysis. The Lancet infectious diseases. 2009;9(3):153-61.
8. Greenaway C, Palayew M, Menzies D. Yield of casual contact investigation by the hour. The International Journal of Tuberculosis and Lung Disease. 2003;7(12):S479-S85.
9. Fok A, Numata Y, Schulzer M, FitzGerald M. Risk factors for clustering of tuberculosis cases: a systematic review of population-based molecular epidemiology studies. The International Journal of Tuberculosis and Lung Disease. 2008;12(5):480-92.
10. Kenyon TA, Valway SE, Ihle WW, Onorato IM, Castro KG. Transmission of multidrug-resistant Mycobacterium tuberculosis during a long airplane flight. New England Journal of Medicine. 1996;334(15):933-8.
11. YIM JJ, Selvaraj P. Genetic susceptibility in tuberculosis. Respirology. 2010;15(2):241-56.
12. Marks SM, Taylor Z, Qualls NL, Shrestha-Kuwahara RJ, Wilce MA, Nguyen CH. Outcomes of contact investigations of infectious tuberculosis patients. American journal of respiratory and critical care medicine. 2000;162(6):2033-8.
13. Society JTCotBT. Control and prevention of tuberculosis in the United Kingdom: code of practice 2000. Thorax. 2000;55(11):887-901.
14. Cabibbe AM, Spitaleri A, Battaglia S, Colman RE, Suresh A, Uplekar S, et al. Application of Targeted Next-Generation Sequencing Assay on a Portable Sequencing Platform for Culture-Free Detection of Drug-Resistant Tuberculosis from Clinical Samples. J Clin Microbiol. 2020;58(10).
15. Hoek KG, Van Rie A, van Helden PD, Warren RM, Victor TC. Detecting drug-resistant tuberculosis: the importance of rapid testing. Molecular Diagnosis & Therapy. 2011;15:189-94.
16. Teixeira L, Perkins M, Johnson J, Keller R, Palaci M, do Valle Dettoni V, et al. Infection and disease among household contacts of patients with multidrug-resistant tuberculosis. The International Journal of Tuberculosis and Lung Disease. 2001;5(4):321-8.
17. Diel R, Loddenkemper R, Meywald-Walter K, Gottschalk R, Nienhaus A. Comparative performance of tuberculin skin test, QuantiFERON-TB-Gold In Tube assay, and T-Spot. TB test in contact investigations for tuberculosis. Chest. 2009;135(4):1010-8.
18. Organization WH. Recommendations for investigating contacts of persons with infectious tuberculosis in low-and middle-income countries: World Health Organization; 2012.
19. Hwang T, Ottmani S, Uplekar M. A rapid assessment of prevailing policies on tuberculosis contact investigation. The International journal of tuberculosis and lung disease. 2011;15(12):1620-3.

For peer review only - http://bmjopen.bmj.com/site/about/guidelines.xhtml

20. Morrison J, Pai M, Hopewell PC. Tuberculosis and latent tuberculosis infection in close contacts of people with pulmonary tuberculosis in low-income and middle-income countries: a systematic review and meta-analysis. The Lancet infectious diseases. 2008;8(6):359-68.
21. Shah NS, Yuen CM, Heo M, Tolman AW, Becerra MC. Yield of contact investigations in households of patients with drug-resistant tuberculosis: systematic review and meta-analysis. Clin Infect Dis. 2014;58(3):381-91.
22. Moher D, Stewart L, Shekelle P. Implementing PRISMA-P: recommendations for prospective authors. Syst Rev. 2016;5:15.
23. Wells GA, Shea B, O'Connell D, Peterson J, Welch V, Losos M, et al. The Newcastle-Ottawa Scale (NOS) for assessing the quality of nonrandomised studies in meta-analyses. Oxford; 2000.
24. Norris JM, Simpson BS, Ball R, Freeman A, Kirkham A, Parry MA, et al. A modified Newcastle-Ottawa scale for assessment of study quality in genetic urological research. European Urology. 2021;79(3):325-6.
25. Schober P, Mascha EJ, Vetter TR. Statistics from a (agreement) to Z (z score): a guide to interpreting common measures of association, agreement, diagnostic accuracy, effect size, heterogeneity, and reliability in medical research. Anesthesia & Analgesia. 2021;133(6):1633-41.
26. Zhai J, Mu W, Si J, Li Y, Zhao C, Shang H, et al. Acupuncture for constipation in patients with stroke: protocol of a systematic review and meta-analysis. BMJ open. 2018;8(3):e020400.

For peer review only

For peer review only - http://bmjopen.bmj.com/site/about/guidelines.xhtml

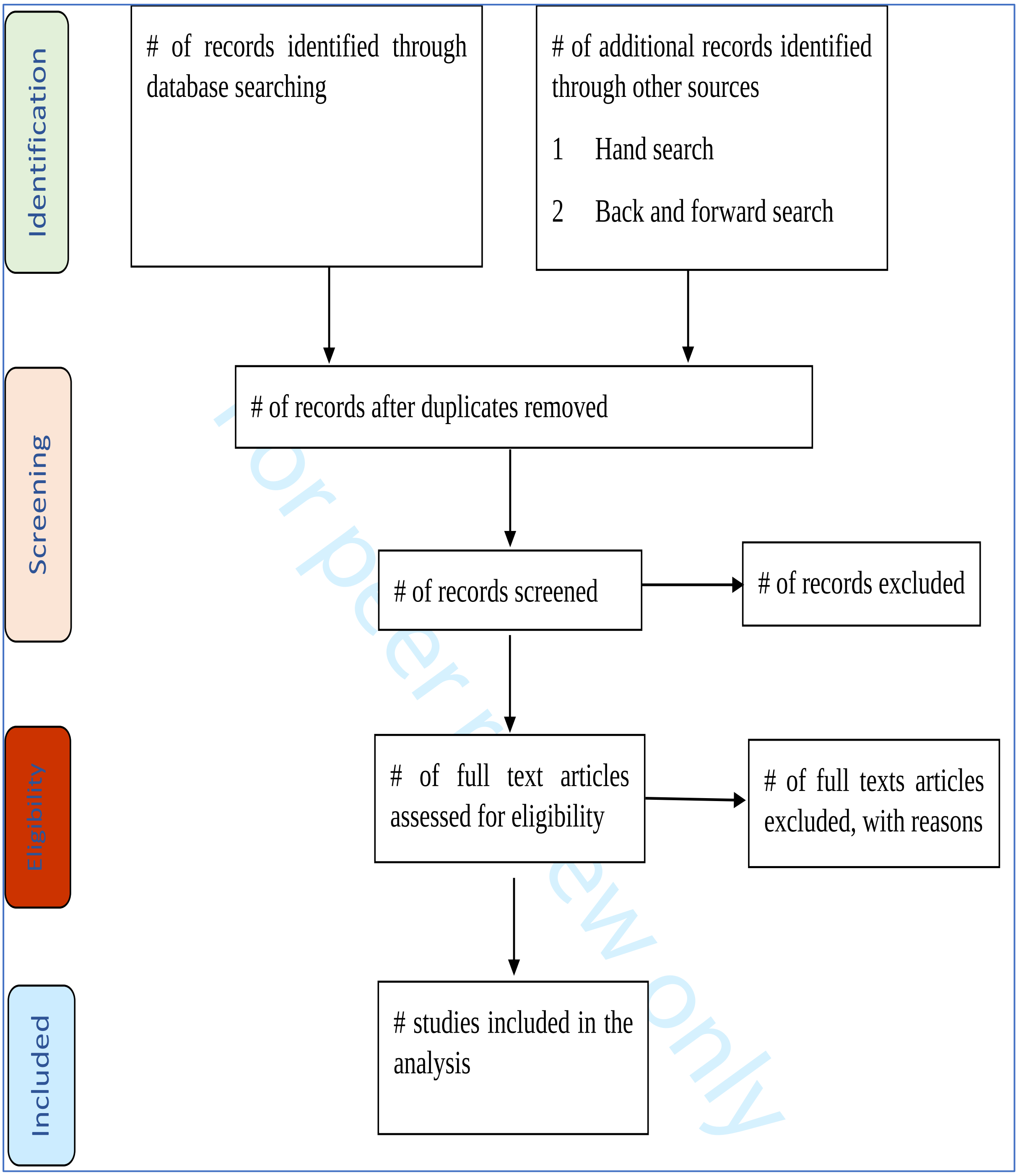

Figure 1: Preferred Reporting Items for Systematic Reviews and Meta-analyses (PRISMA)-2020 flow diagram for the summary of the systematic review study selection process

For peer review only - http://bmjopen.bmj.com/site/about/guidelines.xhtml

Reporting checklist for protocol of a systematic review and meta-analysis.

Based on the PRISMA-P guidelines.

Instructions to authors

Complete this checklist by entering the page numbers from your manuscript where readers will find each of the items listed below.

Your article may not currently address all the items on the checklist. Please modify your text to include the missing information. If you are certain that an item does not apply, please write "n/a" and provide a short explanation.

Upload your completed checklist as an extra file when you submit to a journal.

In your methods section, say that you used the PRISMA-Preporting guidelines, and cite them as:

Moher D, Shamseer L, Clarke M, Ghersi D, Liberati A, Petticrew M, Shekelle P, Stewart LA. Preferred Reporting Items for Systematic Review and Meta-Analysis Protocols (PRISMA-P) 2015 statement. Syst Rev. 2015;4(1):1.

		Reporting Item	Page Number
**Title**			
Identification	#1a	Identify the report as a protocol of a systematic review	1
Update	#1b	If the protocol is for an update of a previous systematic review, identify as such	NA
**Registration**			
	#2	If registered, provide the name of the registry (such as PROSPERO) and registration number	2
**Authors**			
Contact	#3a	Provide name, institutional affiliation, e-mail address of all protocol authors; provide physical mailing address of corresponding author	1
Contribution	#3b	Describe contributions of protocol authors and identify the	10

For peer review only

		guarantor of the review	
**Amendments**			
	#4	If the protocol represents an amendment of a previously completed or published protocol, identify as such and list changes; otherwise, state plan for documenting important protocol amendments	
**Support**			
Sources	#5a	Indicate sources of financial or other support for the review	11
Sponsor	#5b	Provide name for the review funder and / or sponsor	11
Role of sponsor or funder	#5c	Describe roles of funder(s), sponsor(s), and / or institution(s), if any, in developing the protocol	11
**Introduction**			
Rationale	#6	Describe the rationale for the review in the context of what is already known	3-4
Objectives	#7	Provide an explicit statement of the question(s) the review will address with reference to participants, interventions, comparators, and outcomes (PICO)	5
**Methods**			
Eligibility criteria	#8	Specify the study characteristics (such as PICO, study design, setting, time frame) and report characteristics (such as years considered, language, publication status) to be used as criteria for eligibility for the review	7
Information sources	#9	Describe all intended information sources (such as electronic databases, contact with study authors, trial registers or other grey literature sources) with planned dates of coverage	6
Search strategy	#10	Present draft of search strategy to be used for at least one electronic database, including planned limits, such that it could be repeated	6-7
Study records - data management	#11a	Describe the mechanism(s) that will be used to manage records and data throughout the review	9
Study records -	#11b	State the process that will be used for selecting studies (such	8

For peer review only

selection process		as two independent reviewers) through each phase of the review (that is, screening, eligibility and inclusion in meta-analysis)	
Study records - data collection process	#11c	Describe planned method of extracting data from reports (such as piloting forms, done independently, in duplicate), any processes for obtaining and confirming data from investigators	8
Data items	#12	List and define all variables for which data will be sought (such as PICO items, funding sources), any pre-planned data assumptions and simplifications	7
Outcomes and prioritization	#13	List and define all outcomes for which data will be sought, including prioritization of main and additional outcomes, with rationale	7
Risk of bias in individual studies	#14	Describe anticipated methods for assessing risk of bias of individual studies, including whether this will be done at the outcome or study level, or both; state how this information will be used in data synthesis	10
Data synthesis	#15a	Describe criteria under which study data will be quantitatively synthesised	9
Data synthesis	#15b	If data are appropriate for quantitative synthesis, describe planned summary measures, methods of handling data and methods of combining data from studies, including any planned exploration of consistency (such as I2, Kendall's τ)	9
Data synthesis	#15c	Describe any proposed additional analyses (such as sensitivity or subgroup analyses, meta-regression)	10
Data synthesis	#15d	If quantitative synthesis is not appropriate, describe the type of summary planned	NA
Meta-bias(es)	#16	Specify any planned assessment of meta-bias(es) (such as publication bias across studies, selective reporting within studies)	10
Confidence in cumulative evidence	#17	Describe how the strength of the body of evidence will be assessed (such as GRADE)	3

For peer review only

None The PRISMA-P elaboration and explanation paper is distributed under the terms of the Creative Commons Attribution License CC-BY. This checklist can be completed online using https://www.goodreports.org/, a tool made by the EQUATOR Network in collaboration with Penelope.ai

For peer review only
